# The value of high dose tamoxifen in postmenopausal breast cancer patients progressing on standard doses: a pilot study.

**DOI:** 10.1038/bjc.1988.72

**Published:** 1988-03

**Authors:** S. M. Watkins

**Affiliations:** Department of Medical Oncology, Lister Hospital, Stevenage, Herts, UK.


*Br. J. Cancer* (1988), **57**, 320–321 © The Macmillan Press Ltd., 1988

SHORT COMMUNICATION

### The value of high dose tamoxifen in postmenopausal breast cancer patients progressing on standard doses: A pilot study

S.M. Watkins

*Department of Medical Oncology, Lister Hospital, Stevenage, Herts SG1 4AB, UK.*

Tamoxifen is an extremely useful drug in the management of advanced breast cancer. Although it produces remission in only one third of patients, the remarkably low incidence and mild nature of side effects makes it a very acceptable mode of treatment.

Results of attempts to increase response rates by using higher initial doses have been disappointing, (Bratherton *et al.*, 1984; Ortiz de Taranco *et al.*, 1979; Rose *et al.*, 1982) although in one series (Ortiz de Taranco *et al.*, 1979) there were substantially higher rates of stable disease and lower rates of progressive disease in patients receiving 40 mg compared with those on 20 mg daily. Furthermore, there have been occasional case reports of initially responding patients who subsequently relapsed on standard doses of tamoxifen, then going into a second remission when the dose was doubled (Manni & Arafah, 1981; Westerberg *et al.*, 1976). From these observations it appeared that some patients might benefit from achieving blood levels of tamoxifen higher than the usually accepted therapeutic range. Therefore the present pilot study was undertaken to assess the effect of a dose of 90 mg daily in patients who had progressive disease on doses of 20–40 mg daily.

Post-menopausal patients with advanced breast cancer progressing after treatment for at least 10 weeks with tamoxifen at doses of 20–40 mg daily (standard dose), were considered for trial on the high dose. Patients with rapidly progressive disease were excluded and given chemotherapy instead. Seriously ill patients with a life expectancy of less than 3 months were also excluded. Twenty-eight patients aged 49 to 83 (mean 64) were included. They were 5 months to 35 years post-menopausal (mean 15 years). Six had had prior treatment with other endocrine manipulations, and 9 had had chemotherapy. Standard dose tamoxifen was given for a minimum of 10 weeks, and responses were assessed according to UICC criteria (Hayward *et al.*, 1978). On standard dose tamoxifen there had been 10 cases of progressive disease; four patients went into complete remission, there were 4 partial remissions, and 10 with stable disease: all these 18 patients subsequently developed progressive disease. When the patients showed evidence of primary or secondary progression, the dose of tamoxifen was increased to 30 mg tds. Patients were reviewed at least once a month by SMW. X-rays were independently assessed by a consultant radiologist. Initially abnormal chest X-rays and liver function tests were repeated every month and skeletal X-rays every 6–8 weeks, or sooner if there was any clinical indication.

The tamoxifen used in this study was Nolvadex (ICI plc, UK). Serum levels of tamoxifen were measured before and 8 weeks after starting the high dose. The tamoxifen levels were assayed by ICI Pharmaceuticals using the HPLC method. Aliquots of serum (1.0 ml) were spiked with internal standard (N-dipropyl analogue of tamoxifen). Following the addition of 1.0 M phosphate buffer, pH 7 (1.0 ml) the samples were extracted with 1.5% (v/v) amyl alcohol in hexane (5 ml). After tumble shaking for 1 h and centrifugation to separate the phases, a portion (4.5 ml) of the organic phase was transferred to a clean tube and reduced to dryness at room temperature under a stream of oxygen-free nitrogen. The dry residue was redissolved in HPLC eluent (500 $\mu$l). Aliquots of the reconstituted extracts (typically 50 $\mu$l) were separated on a 5 $\mu$ Zorbax ODS column (10 cm × 4.6 mm) eluted at a flow rate of 1.5 ml min$^{-1}$, with tetrahydrofuran-acetonitrile-water-ammonia (sp. gr. 0.88) 15:70:15:0.4 by volume. The tamoxifen was detected in the column eluent by its uv absorption at 240 nm.

Response was assessed according to UICC criteria (Hayward *et al.*, 1978). There were two partial remissions lasting 5 and 8 months, and 15 patients with stable disease lasting from three to 19 months (median 5 months). Many of these stable patients had considerable symptomatic relief on the higher dose. There was no correlation between response to standard dose and high dose tamoxifen.

With regard to the various sites of disease, the best response rates were seen in primary lesions and lymph node metastases (complete plus partial regression 3/8 and 5/8 respectively). Stable disease was frequently seen in soft tissue lesions (8/11 plus 1/11 partial remission). Bony lesions also appeared to remain static for long periods (12/19 stable disease), but in only one patient was overall stable disease assessed on bony lesions alone.

Mild side-effects (lethargy, tiredness, loss of taste and hot flushes) were reported in 4 patients, but no-one withdrew from the trial on this account. There was no evidence of retinopathy.

The steady state serum concentrations of tamoxifen rose when patients took the higher dose, but levels varied widely, and there was no correlation between response and serum levels of the drug (Figure 1).

This study shows that many post-menopausal women with advanced breast cancer, progressing in spite of treatment with standard dose tamoxifen, benefit from receiving the drug in high doses (90 mg daily). Although there were only occasional remissions, there was stabilisation of disease (frequently with symptomatic improvement) in over half the patients, and as side-effects were minimal, this resulted in good quality life, often for many months. A comparable observation was made by Stewart *et al.* (1982) who showed that although increasing the dose of tamoxifen from 20 mg to 40 mg daily in patients with progressive disease did not produce objective responses, yet a quarter of these patients had stable disease for up to 15 months.

In our patients, the best responses to high dose tamoxifen were seen in primary tumours and lymph node metastases; however, the numbers are too small for statistical analysis. Stable disease was observed in two thirds of bony metastases, although in only one patient (number 14) was overall stable disease assessed on bony lesions alone. Radiological changes in metastatic bone cancer are usually slow and hence difficult to interpret. Indeed, it has been suggested that in some cases of apparently static bone disease, there may actually be some tumour response, in spite of the absence of discernable radiological evidence of healing (Coleman & Rubens, 1987).

Received 30 October 1987; and in revised form, 7 January 1988.

**Figure 1** Blood levels of tamoxifen in patients on standard dose and high dose tamoxifen. Correlation of steady state values with dose in individual patients and response at the higher dose. (●) Progressive disease; (○) stable disease; (□) partial response.

Although there does appear to be a change in the rate of progression of disease in some patients after dose increases, in our patients, as in the series of Bratherton *et al.* (1984), there was no correlation between serum levels and clinical benefit.

The mechanism of the improved efficacy of tamoxifen at high doses is not clear. The oestrogen-reversible inhibitory effect of tamoxifen on cell proliferation in ER-positive cell lines *in vitro* is clearly seen at the low concentrations of tamoxifen corresponding to the usual therapeutic steady state blood levels achieved on standard doses of the drug (Reddel *et al.*, 1985).

However, oestrogen-irreversible cytotoxicity has been demonstrated *in vitro* in both ER-positive and ER-negative cell lines at high concentrations of tamoxifen (albeit higher than the blood levels achieved in this study), suggesting that under these circumstances growth inhibition and cytotoxicity may be mediated by mechanisms independent of the anti-oestrogen effect (Reddel *et al.*, 1985). There is *in vitro* evidence that tamoxifen and its metabolites in high concentrations inhibit protein kinase C, an enzyme which mediates signals for cellular proliferation (O'Brian *et al.*, 1986). On the other hand, Gulino *et al.* (1986) felt that protein kinase C inhibition was unimportant and have suggested that the interaction between anti-oestrogen and calmodulin may be responsible for mediating drug-induced, oestrogen-independent inhibition of breast cancer cell growth, possibly by causing intracellular accumulation of cAMP. The relevance of these *in vitro* findings to the observations made in the present study remain to be elucidated.

Although in our study, increasing the dose of tamoxifen to 90 mg daily produced only two partial remissions, nevertheless, the high incidence and sometimes prolonged duration of stable disease with minimal side-effects and good quality of life, makes this a useful and highly acceptable approach in cases of primary and secondary failure of tamoxifen treatment, and well worth trying before switching to other forms of endocrine therapy or chemotherapy, all of which have more serious side-effects.

My thanks to Dr W.P. Abram of Belvoir Hospital, Belfast, for contributing three patients to this study; to Professor Adrian Harris of the University of Newcastle-upon-Tyne for his interest and advice; and to Imperial Chemical Industries, PLC for the supply of Nolvadex, for the plasma level measurements, and for continuing advice and cooperation.

### References

BRATHERTON, D.G., BROWN, C.H., BUCHANAN, R. & 4 others (1984). A comparison of two doses of tamoxifen (Nolvadex) in postmenopausal women with advanced breast cancer: 10 mg bd versus 20 mg bd. *Br. J. Cancer*, **50**, 199.

COLEMAN, R.E. & RUBENS, R.D. (1987). The clinical course of bone metastases from breast cancer. *Br. J. Cancer*, **55**, 61.

GULINO, A., BARRERA, G., VACCA, A. & 5 others (1986). Calmodulin antagonism and growth-inhibiting activity of triphenylethylene antiestrogens in MCF-7 human breast cancer cells. *Cancer Res.*, **46**, 1.

HAYWARD, J.L., RUBENS, R.D., CARBONE, P.P., HEUSON, J.-C., KUMAOKA, S. & SEGALOFF, A. (1978). Assessment of response to therapy in advanced breast cancer. *Europ. J. Cancer*, **14**, 1291.

MANNI, A. & ARAFAH, B.M. (1981). Tamoxifen-induced remission in breast cancer by escalating the dose to 40 mg daily after progression on 20 mg daily: A case report and review of the literature. *Cancer*, **48**, 873.

O'BRIAN, C.A., LISKAMP, R.M., SOLOMON, D.H. & WEINSTEIN, I.B. (1986). Triphenylethylenes: A new class of protein kinase C inhibitors. *J. Natl Cancer Inst.*, **76**, 1243.

ORTIZ DE TARANCO, A.V., DONNAY CANDIL, O., BAENA HERRERA, L.F., GUARDIOLA DELEGIDO, L. & RUBIO MERINERO, D. (1979). Tratamiento de carcinoma mamario (estadio IV), en 78 enfermas postmenopausicas, mediante antiestrogenos (Nolvadex). *Oncologia*, **80**, 49.

REDDEL, R.R., MURPHY, L.C., HALL, R.E. & SUTHERLAND, R.L. (1985). Differential sensitivity of human breast cancer cell lines to the growth-inhibitory effect of tamoxifen. *Cancer Res.*, **45**, 1525.

ROSE, C., THEILADE, K., BOESEN, E. & 5 others (1982). Treatment of advanced breast cancer with tamoxifen: Evaluation of the dose-response relationship at two dose levels. *Breast Cancer Res. Treat.*, **2**, 395.

STEWART, J.F., MINTON, M.J. & RUBENS, R.D. (1982). Trial of tamoxifen at a dose of 40 mg daily after disease progression during tamoxifen therapy at a dose of 20 mg daily. *Cancer Treat. Rep.*, **66**, 1445.

WESTERBERG, H., NORDENSKJÖLD, B., DE SCHRYVER, A. & NOTTER, B. (1976). Anti-oestrogen therapy of advanced mammary carcinoma. *Acta Radiol. Therap. Phys. Biol.*, **15**, 513.


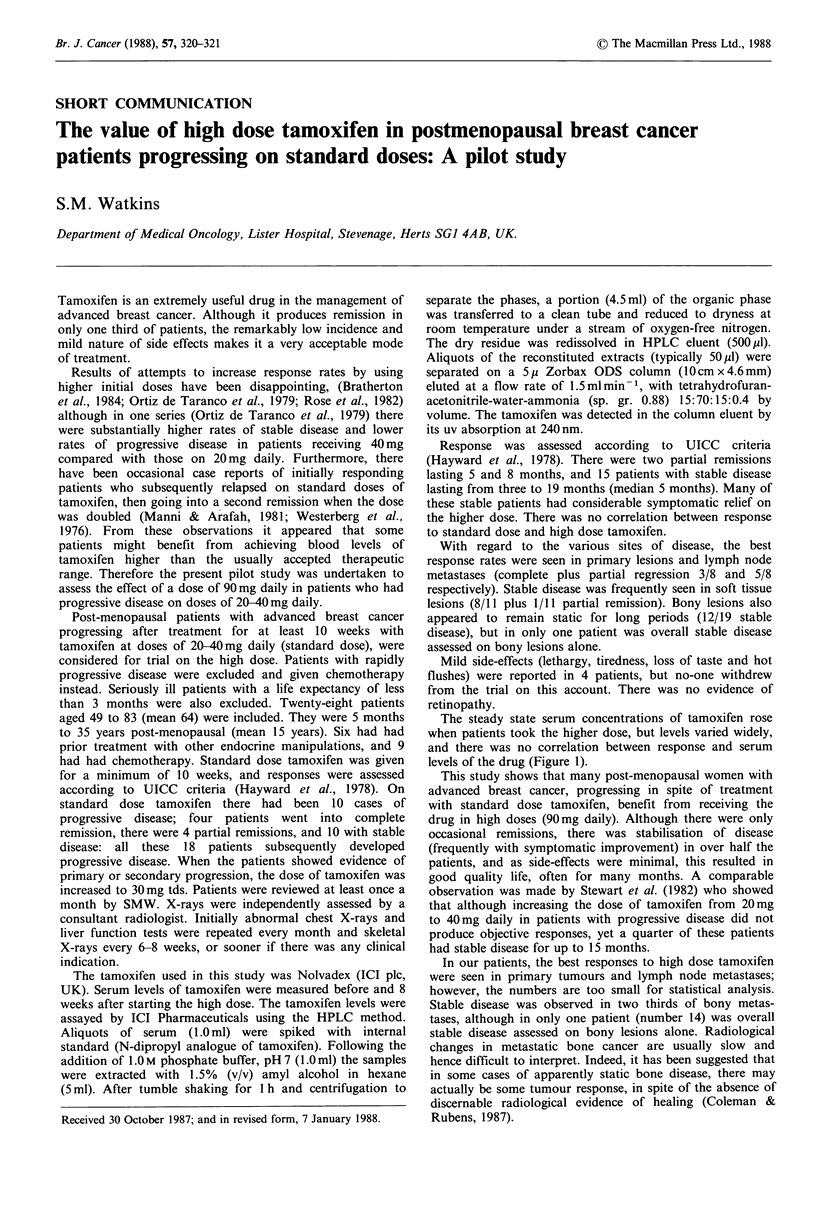

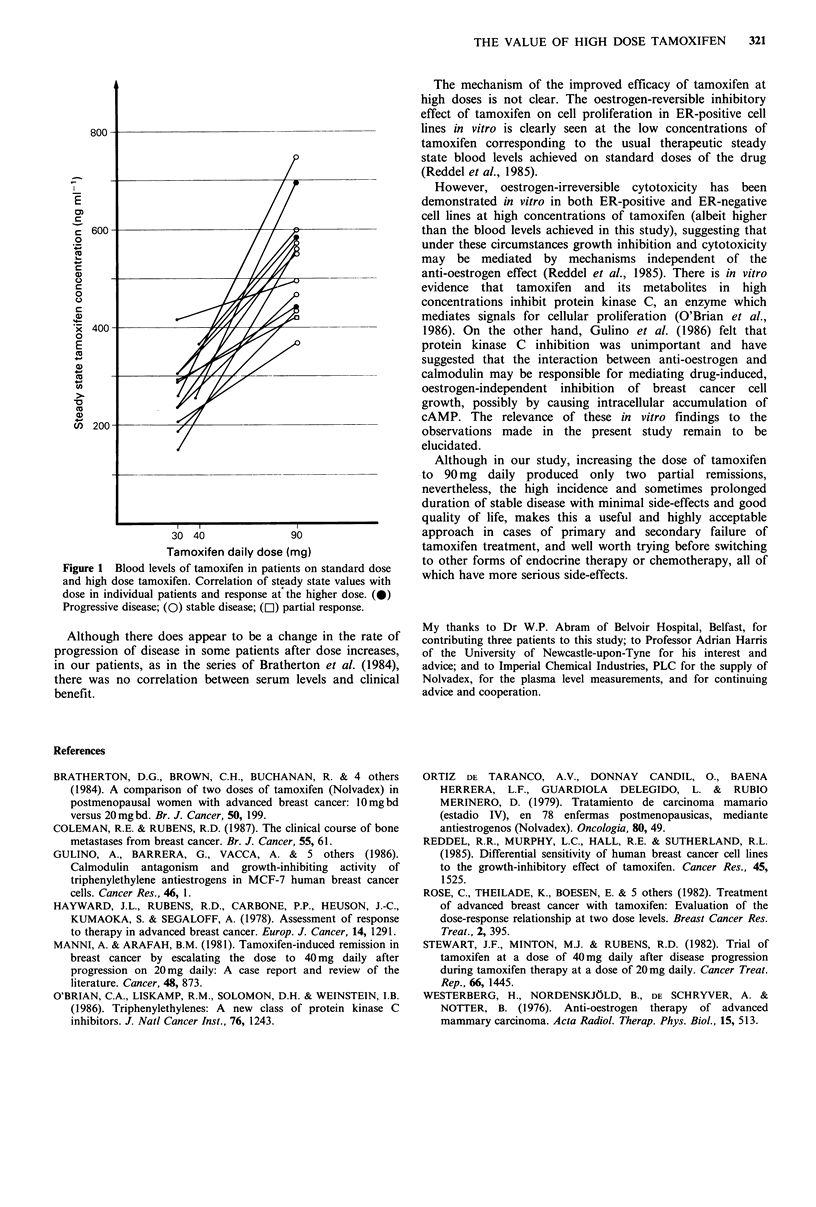


## References

[OCR_00233] Bratherton D. G., Brown C. H., Buchanan R., Hall V., Kingsley Pillers E. M., Wheeler T. K., Williams C. J. (1984). A comparison of two doses of tamoxifen (Nolvadex) in postmenopausal women with advanced breast cancer: 10 mg bd versus 20 mg bd.. Br J Cancer.

[OCR_00239] Coleman R. E., Rubens R. D. (1987). The clinical course of bone metastases from breast cancer.. Br J Cancer.

[OCR_00249] Hayward J. L., Rubens R. D., Carbone P. P., Heuson J. C., Kumaoka S., Segaloff A. (1978). Assessment of response to therapy in advanced breast cancer. A project of the programme on clinical oncology of the International Union against Cancer, Geneva, Switzerland.. Eur J Cancer.

[OCR_00253] Manni A., Arafah B. M. (1981). Tamoxifen-induced remission in breast cancer by escalating the dose to 40 mg daily after progression on 20 mg daily: a case report and review of the literature.. Cancer.

[OCR_00259] O'Brian C. A., Liskamp R. M., Solomon D. H., Weinstein I. B. (1986). Triphenylethylenes: a new class of protein kinase C inhibitors.. J Natl Cancer Inst.

[OCR_00271] Reddel R. R., Murphy L. C., Hall R. E., Sutherland R. L. (1985). Differential sensitivity of human breast cancer cell lines to the growth-inhibitory effects of tamoxifen.. Cancer Res.

[OCR_00283] Stewart J. F., Minton M. J., Rubens R. D. (1982). Trial of tamoxifen at a dose of 40 mg daily after disease progression during tamoxifen therapy at a dose of 20 mg daily.. Cancer Treat Rep.

[OCR_00289] Westerberg H., Nordenskjöld B., de Schryver A., Notter G. (1976). Anti-oestrogen therapy of advanced mammary carcinoma.. Acta Radiol Ther Phys Biol.

